# Renal Angiomyolipoma Mimicking a Well-Differentiated Retroperitoneal Liposarcoma

**DOI:** 10.1155/2020/8812057

**Published:** 2020-09-24

**Authors:** Honoka Fuse, Masaya Ito, Kosuke Takemura, Shuzo Ikuta, Toru Motoi, Tomotake Okuma, Madoka Kataoka, Fumitaka Koga

**Affiliations:** ^1^Department of Urology, Tokyo Metropolitan Cancer and Infectious Diseases Center Komagome Hospital, Tokyo, Japan; ^2^Department of Radiology, Tokyo Metropolitan Cancer and Infectious Diseases Center Komagome Hospital, Tokyo, Japan; ^3^Department of Pathology, Tokyo Metropolitan Cancer and Infectious Diseases Center Komagome Hospital, Tokyo, Japan; ^4^Department of Musculoskeletal Oncology, Tokyo Metropolitan Cancer and Infectious Diseases Center Komagome Hospital, Tokyo, Japan

## Abstract

A 37-year-old Burmese woman presented with an incidentally found retroperitoneal fat-containing tumor. The tumor was 9 cm in the longest diameter, surrounding the right kidney, and composed of homogenous adipose tissue with thickened septum-like structures and spotty nonadipose structures, which were enhanced on contrast-enhanced computed tomography and magnetic resonance imaging. The tumor did not show either a beak sign or synchronous angiomyolipoma-like lesion in the kidneys. The tumor had irregular septa, thin blood vessels, and spotty small soft-tissue nodules. The tumor did not contain any heterogeneously enhanced solid lesions suspicious for dedifferentiated liposarcomas. Based on these imaging findings, a clinical diagnosis of a well-differentiated liposarcoma was made. Under the consensus of a multidisciplinary cancer board, she was recommended to undergo core-needle biopsy to confirm the clinical diagnosis. However, she declined to undergo biopsy for financial reasons. She underwent kidney-sparing retroperitoneal tumor resection. Histopathologically, the tumor was an angiomyolipoma with positive immunostaining for HMB45 and Melan A. The present case suggests the importance of core-needle biopsy prior to surgical intervention for retroperitoneal fat-containing tumors.

## 1. Introduction

Several types of fat-containing tumors arise in the retroperitoneum. Of these, the differential diagnosis between well-differentiated liposarcomas (WDLs) and large renal angiomyolipomas (AMLs) is sometimes challenging [1, 2]. Although large AMLs are generally exophytic and extend into the perinephric space, both WDLs and large renal AMLs might present as large fat-containing tumors attached to the kidneys. Despite the radiographic similarities, treatment options for these tumors are quite different. The standard of care for WDLs is surgical resection because of the possible acquisition of an enhanced aggressive phenotype such as that of dedifferentiated liposarcomas (DDLs) [3], whereas patients with AMLs have several treatment options including active surveillance, selective arterial embolization, ablative therapies, surgical treatment, and the administration of mammalian target of rapamycin (mTOR) inhibitors [4]. Thus, it is important to establish accurate diagnoses for the appropriate management of retroperitoneal fat-containing tumors. Here, we report a case of a renal AML mimicking a retroperitoneal WDL.

## 2. Case Presentation

**Figure 1:**
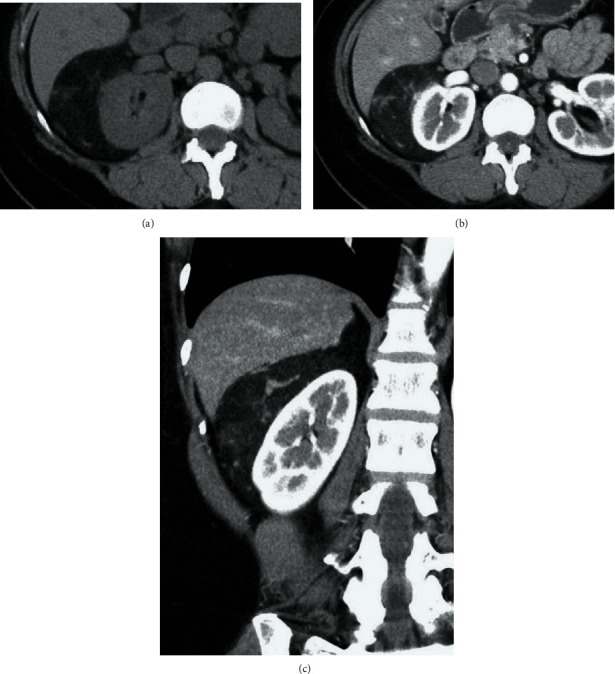
Computed tomography findings. (a) A plain axial section. The tumor was mainly composed of adipose tissue with thick irregular septum-like structures. (b) A contrast-enhanced axial section. The septum-like structures showed early enhancement. (c) A contrast-enhanced coronal section. There was no beak sign or sharp beak shape arising from the kidneys.

**Figure 2:**
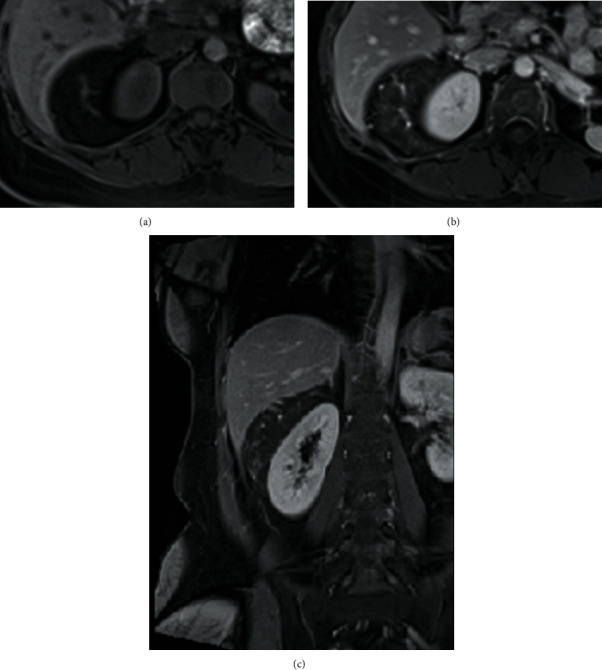
Magnetic resonance imaging findings. (a) Fat-suppressed T1-weighted scans. The tumor was mainly composed of a homogenously low-intensity area, including a weakly high-intensity area with thick irregular septum-like structures. (b) Dynamic contrast-enhanced image. The septum-like structures showed strong enhancement. There was no beak sign or sharp beak shape arising from the kidneys. (c) Coronal section of dynamic contrast-enhanced image. The tumor surrounded the right kidney with no beak sign.

**Figure 3:**
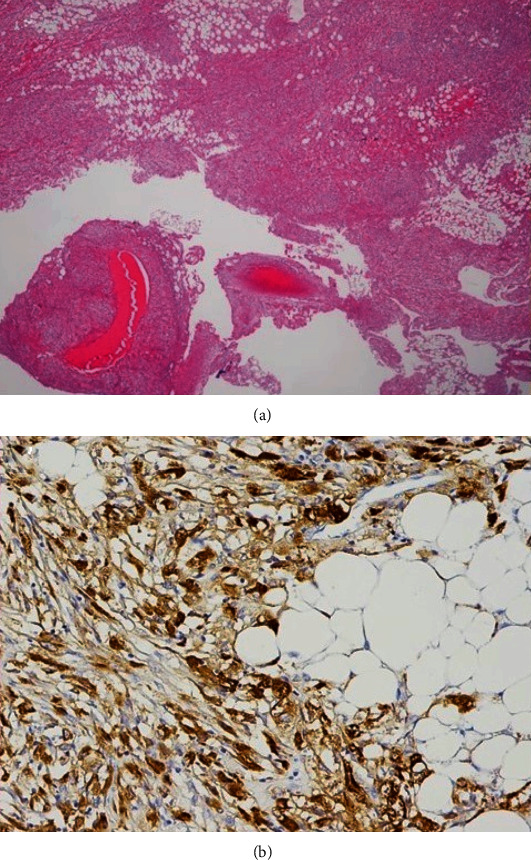
Histopathological findings. (a) Hematoxylin–eosin stain (×40). The tumor consisted of mature adipose tissue, smooth muscle cells, and thick-walled abnormal vessels. (b) Immunostaining for HMB45, a marker of angiomyolipoma, was positive in the tumor cells (×200).

A 37-year-old Burmese woman presented with an incidental retroperitoneal tumor. Contrast-enhanced computed tomography (CT) revealed a tumor measuring 9 cm in the longest diameter surrounding the right kidney (Figure 1). The tumor consisted of adipose tissue and thickened irregular septum-like structures showing early enhancement on CT. The tumor did not demonstrate a “beak sign,” a sharp beak shape arising from the kidneys, nor an “embedded organ sign,” encasement of the organ of origin in the tumor, both of which are often observed in cases of renal AMLs [5]. The tumor had thin blood vessels and spotty small soft-tissue nodules. On the other hand, the tumor did not present with hemorrhage, aneurysm, or intratumoral calcification. There were no other synchronous AML-like lesions in the ipsilateral or contralateral kidney, nor did tumor vessels extend through the renal parenchyma. The tumor contained no solid lesions suspicious for DDLs. On fat-suppressed T1-weighted magnetic resonance imaging (MRI), the tumor had a homogenously low-intensity area containing a weakly high-intensity area with a thick irregular septum-like structure (Figure 2(a)) exhibiting strong enhancement (Figure 2(b)). Her case was evaluated in a cancer board composed of urologists, soft-tissue oncologists, radiation oncologists, radiologists, and pathologists, wherein the consensus of a clinical diagnosis of a retroperitoneal WDL was reached. Even though we strongly recommended core-needle biopsy before surgery, she declined this and desired upfront surgical resection of the tumor for financial reasons. She subsequently underwent kidney-sparing retroperitoneal tumor resection through a minimum-incision endoscopic surgery [6], and she was discharged without complications. Histopathological examination revealed a heterogeneous tumor consisting of mature adipose tissue, spindle and epithelioid smooth muscle cells, and abnormal thick-walled blood vessels (Figure 3(a)). There were no atypical cells corresponding to the diagnosis of malignancy. Immunohistochemically, the tumor cells tested strongly and diffusely positive for HMB45 (Figure 3(b)). The tumor cells also tested positive for Melan A, smooth muscle actin, and S-100. Consequently, a definite diagnosis of AML was established.

## 3. Discussion

We encountered a case of a renal AML mimicking a WDL. It is occasionally challenging to differentiate AMLs from WDLs because both tumors might appear as similar large fat-containing perinephric masses. To distinguish them radiologically, it has been reported that renal AMLs often exhibit characteristic findings of a “beak sign” and “embedded organ sign” on CT and MRI [5]. Additionally, AMLs have been reported to possess identical features of the presence of synchronous AML-like lesions in other areas and the presence of hemorrhage, aneurysm, and tumor vessels extending through the renal parenchyma [7]. In the present case, none of these features of AMLs were observed.

WDLs and DDLs are known to originate from retroperitoneal soft tissue and harbor adipose tissue and thickened irregular septum-like structures exhibiting strong enhancement [5, 8]. Although WDLs and DDLs have several common features, including irregular septa, thin blood vessels, and intratumoral calcification [7], distinct differences lie in that DDLs are frequently accompanied by solid nodules that are strongly enhanced on contrast-enhanced CT and are completely independent from fatty elements [7, 9], while WDLs exhibit features of soft-tissue nodules with weaker contrast enhancement [10]. In the present case, a clinical diagnosis of a retroperitoneal WDL was established because most features of WDLs were observed apart from intratumoral calcification, whereas none of the features of DDLs were observed. Hence, we considered that a WDL was the most probable diagnosis and that core-needle biopsy would provide additional information before surgical intervention. If a tumor biopsy had been performed and an AML had been suggested before the surgery, she could have chosen a less invasive treatment option, including selective arterial embolization, ablative therapies, and the administration of mTOR inhibitors [4]. Active surveillance could also have been an option, although large AMLs harbor the risk of spontaneous rupture [11, 12].

## 4. Conclusions

We encountered a case of a renal AML mimicking a WDL on imaging examinations. The important role of core-needle biopsy is suggested for accurate clinical diagnosis and proper management of retroperitoneal fat-containing tumors.
